# Genome Sequence of Methylotrophic Azospirillum sp. Strain B2, Isolated from a Raised Sphagnum Bog

**DOI:** 10.1128/genomeA.00492-18

**Published:** 2018-06-07

**Authors:** Denis S. Grouzdev, Ekaterina N. Tikhonova, Maria S. Krutkina, Irina K. Kravchenko

**Affiliations:** a Institute of Bioengineering, Research Center of Biotechnology of the Russian Academy of Sciences, Moscow, Russia; b Winogradsky Institute of Microbiology, Research Center of Biotechnology of the Russian Academy of Sciences, Moscow, Russia

## Abstract

Azospirillum sp. strain B2 is a soil bacterium which was originally isolated from the Sosvyatskoe raised Sphagnum bog in Russia. Here, we present the approximately 8-Mb draft genome sequence of Azospirillum sp. B2, with the aim of providing insight into the genomic basis of its ecological success in peatland settings.

## GENOME ANNOUNCEMENT

Bacteria of the genus Azospirillum are found primarily in terrestrial habitats, where they colonize roots of important cereals and other grasses and promote plant growth by several mechanisms, including nitrogen fixation and phytohormone secretion. Azospirillum sp. strain B2 is a Gram-negative rod-shaped member of the Alphaproteobacteria. First, Azospirillum sp. B2 was revealed to be a nitrogen-fixing component of a stable methane-oxidizing consortium from acidic Sphagnum-dominated peat soil (1) and then isolated as a pure culture (2). The ecological success of Azospirillum sp. B2 in the peat soil may be related to the presence in its genome of genetic systems which allow it to oxidize and assimilate C_1_ compounds. Based on its 16S rRNA gene sequence, Azospirillum sp. B2 shares high similarity with Azospirillum humicireducens strain SgZ-5^T^ (98.8% similarity).

Genomic DNA was extracted according to the method of Wilson (3), with minor modifications. The DNA was sonicated on a Covaris S2 device to an average fragment size of 250 bp. Libraries were constructed with the NEBNext DNA library prep reagent set for Illumina, according to the protocol for the kit. The libraries were sequenced using an Illumina HiSeq 1500 platform with 150-bp read length. Genome assembly was performed using SPAdes version 3.10.0 (4). A total of 99 contigs longer than 500 bp with a length of 7,997,491 bp were obtained. The *N*_50_ value of the contigs was 246,279 bp. The average G+C content of the genome of Azospirillum sp. B2 was 67.8%. All contigs were submitted to GenBank, where gene annotation was implemented using the NCBI Prokaryotic Genome Annotation Pipeline (PGAP) (5). Among the 7,059 genes predicted by PGAP, 6,821 were protein-coding genes, 66 coded for tRNAs, and 4 belonged to noncoding RNAs (ncRNAs).

The genome encodes the Embden-Meyerhof-Parnas glycolytic pathway and the tricarboxylic acid cycle. The *mxaFI* genes encoding methanol dehydrogenase were absent, but a homologous *xoxF* gene was detected. The genes encoding enzymes involved in the biosynthesis of tetrahydromethanopterin (H_4_MPT) (formaldehyde oxidation) and genes encoding NAD-linked formate dehydrogenase (*fdsABG*) were identified. Key genes for the ribulose monophosphate pathway and the serine cycle were absent; however, genes associated with the Calvin-Benson-Bassham pathway of C_1_ assimilation were present.

Strain B2 was able to grow with ammonium, nitrate, or dinitrogen gas as a nitrogen source. Genes responsible for ammonium uptake (*amtB*) and assimilation (*glnA*, *gltB*, and *ald*) were detected. A full complement of genes for dinitrogen fixation (*nif*) were also found. In addition, genes for nitric oxide reduction (*norB* and *norC*) were identified.

### Accession number(s).

This whole-genome shotgun project has been deposited at DDBJ/ENA/GenBank under the accession no. PDKW00000000. The version described in this paper is version PDKW01000000.
